# Our search of immune invaders in the aged lacrimal gland

**DOI:** 10.18632/aging.204651

**Published:** 2023-08-11

**Authors:** Claudia M. Trujillo-Vargas, Cintia S. de Paiva

**Affiliations:** 1 Department of Ophthalmology, Baylor College of Medicine, Houston, TX 77030, USA; 2 Grupo de Inmunodeficiencias Primarias, Facultad de Medicina, Universidad de Antioquia, UdeA, Medellín, Colombia

**Keywords:** lacrimal gland, aging, ectopic lymphoid structures, immune invasion

Imagine that we are in a place where “we see things that you need not see and we be places that you need not be”, paraphrasing the song of "Men in Black", one of our favorite movies ever. This is the landscape of some human lacrimal glands with aging. An exocrine organ that has lifelong lubricated our most precious connection tool to the universe, the eyes, without apparent microbial insult, gradually become invaded by aberrant immune cells. These highly activated lymphocytes potentially support the development of auto-antibodies, powerful facilitators of cell destruction in the lacrimal tubuloacinar structure. Cell damage and fibrosis follow immune invasion and become important players of the so-called dry eye disease, a frequent although very heterogeneous condition with aging, especially in women. Burning and redness in the eyes, grittiness and blurry vision make life miserable and currently, eye drops with a variety of lubricant components and in the most severe cases, immunosuppressors, are the only therapies approved for this disease.

For several years our group has committed to study the immunopathological changes in the lacrimal gland with aging. With the scarcity of human specimens, our research has focused on the characterization of the lacrimal gland lymphocyte invasion in aged mice. We have demonstrated that aged lacrimal glands are infiltrated not only by highly differentiated B but also T cells. This landscape is associated with increased ocular surface dysfunction [1, 2]. In the search of mechanisms that can counteract the effects of the overwhelming immune infiltration, we started characterizing one of the main players of immune tolerance, the thymic-derived T regulatory cells (Tregs). Tregs possess the ability to hamper the activation of conventional T cells through a variety of mechanisms [3]. But let’s stay within the frame of "Men in Black", where human-like beings turn into aliens on multiple occasions: similar scenery happens with the tolerogenic Tregs in the aged gland. They express all the markers that support their suppressive function but paradoxically, seem highly differentiated and infiltrative, actively produce inflammatory cytokines, possess defective suppressive capabilities and more importantly, recapitulate lacrimal gland pathology when adoptively transferred to immunodeficient recipients [3, 4]. Moreover, in the aged lacrimal gland, Tregs are surrounded by CD4+ T cells that are highly differentiated towards the effector Th1 and Th17 phenotype and have features compatible with exhaustion and immunopathology [3]. These characteristics seem to make it more difficult for Tregs to exert their immunosuppressive function. Another interesting signature of conventional CD4+ T cells in the aged lacrimal gland is their enrichment in cells with a naïve phenotype. Interestingly, we have also observed increased IgD+ B cells in the aged lacrimal gland, hallmarks of naïve B cells [1]. This finding could indicate a nurturing but aberrant environment for the recruitment and differentiation of inexperienced naïve cells in the gland, outside the sites with the microarchitecture and immune checkpoints to develop this function: the secondary lymphoid organs. These peculiar lymphocytic aggregates that have been called ectopic lymphoid structures are not uncommon in other aged tissues [1]. One might also think that it is beneficial for the immune system to recruit highly activated cells to peripheral tissues such as the lacrimal gland. However, highly differentiated and activated but otherwise exhausted T cells have demonstrated to be inefficient to attack virus or cancer cells [5].

Another aspect to consider is the implications of an immune cell invasion in the lacrimal gland. According to the Oxford dictionary, an invasion is an “unwelcome intrusion into another's domain.” Are immune cells really unwelcome in the lacrimal gland? Strictly speaking, the answer is no, they are very welcome! Contrary to the immune landscape of the cornea, which is considered an immune privileged tissue, the lacrimal gland is highly vascularized and has a low but constant influx of immune cells since early in life, likely exerting a surveillance function. Moreover, dendritic cells and other antigen presenting cells are sporadically found in proximity to the immune aggregates in the interstitium [6]. However, lymphocytic infiltration steadily increases with age together with the presence of fibrosis, duct pathology and atrophy in the gland [7]. This juxtaposes a decreased number of antigen presenting cells in the aged gland, which contribute to the bizarre immune environment [2].

Now, let’s remember one of the most potent weapons to attack the feared aliens in "Men in Black": the noisy cricket. Well, we are in the search of those potential “noisy crickets” in the aged gland: i.e., effective but molecular-scale therapies to fight against the pathological immune infiltration. In order to do this, we have started characterizing the immune-related differentially expressed genes in the aged gland. These studies will support the development of biological targets of drug discovery. We observed that Tregs in the aged gland expressed high levels of *Il1r2*, *CD81*, and *Tbx21*, among others. *Tbx21* is also highly expressed by conventional T cells [3] and increased expression of *CD79a/b* is observed in activated B cells [1]. A systematic review of biological therapies for Sjögren’s syndrome, an autoimmune disease that represent “the tip of the iceberg”, i.e., the most severe presentation of the sicca syndrome in exocrine glands, described 25 promising ongoing clinical trials that target lymphocyte signaling and function and other inflammatory pathways [8]. However, biologic costs still represent an obstacle for patient accessibility and therefore, we are also investigating the role of the gut microbiota in the development of ocular barrier disruption in mice [9]. These studies would potentially deliver beneficial microbial consortia to treat dry eye disease in humans, an approach that might be more cost effective. Whether these therapies may work to impede lymphocyte infiltration in the aged lacrimal gland in humans is still not known.

With the accelerated changes in human behavior in the last decade, which expose us to heavily polluted environments and also make us dramatically dependent on screens, we anticipated a steady increment in highly injured lacrimal glands in the elderly. This represents an enriched environment for the development of ectopic lymphoid structures, likely causing an elevation in the prevalence of dry eye disease in the population. Unquestionably, more than “fancy sunglasses” would be needed to hinder the “carbonizing” immune damage in the gland. Thus, Yes! We certainly need to protect our lacrimal glands from the scum of our own immune universe!
